# Fermented herbal formula KIOM-MA-128 protects against acute colitis induced by dextran sodium sulfate in mice

**DOI:** 10.1186/s12906-017-1855-4

**Published:** 2017-07-05

**Authors:** Dong-Gun Kim, Mi-Ra Lee, Jae-Myung Yoo, Kwang-Il Park, Jin-Yeul Ma

**Affiliations:** Korea Medicine (KM)-Application Center, Korea Institute of Oriental Medicine, 70 Cheomdan-ro, Dong-gu, Daegu, 41062 Republic of Korea

**Keywords:** Ulcerative colitis, Herbal medicine, Inflammation, Tight junction, Colon, KIOM-MA-128

## Abstract

**Background:**

Colitis is a well-known subtype of inflammatory bowel disease and is caused by diverse factors. Previous research has shown that KIOM-MA elicits anti-inflammatory and anti-allergic effects on various diseases. KIOM-MA-128, our novel herbal formula, was generated from KIOM-MA using probiotics to improve the therapeutic efficacy. We investigated whether KIOM-MA-128 has protective activity in a mouse model of acute colitis induced by dextran sodium sulfate (DSS).

**Methods:**

Colitis was induced by DSS administered to ICR mice in drinking water. KIOM-MA-128 (125 or 250 mg/kg) was orally administered once per day. The body weights of the mice were measured daily, and colonic endoscopies were performed at 5 and 8 days. Colon length as well as histological and cytokine changes were observed at the end of drug administration.

**Results:**

KIOM-MA-128 has pharmacological activity in an acute colitis model. KIOM-MA-128 reduced the loss of body weight and disease activity index (DAI) and inhibited the abnormally short colon lengths and the colonic damage in this mouse model of acute colitis. Moreover, KIOM-MA-128 suppressed pro-inflammatory cytokine expression and maintained the integrity of the tight junctions during DSS-induced colitis.

**Conclusion:**

The results indicated that KIOM-MA-128 protects against DSS-induced colitis in mice and suggested that this formula might be a candidate treatment for inflammatory bowel disease (IBD).

## Background

Ulcerative colitis (UC) and Crohn’s disease (CD) are subtypes of inflammatory bowel disease (IBD) [1]. Symptoms of UC and CD might include abdominal pain, diarrhea and/or fever [2], and UC and CD are caused by environmental interactions, genetic factors, and lifestyle factors [3]. Therapeutic approaches to treating colitis include administration of anti-inflammatory drugs and immunomodulators, surgery, and therapies focused on controlling the immune cell-response cytokine pathway; however, treatments are limited because of several severe side effects such as allergies and lymphoma [4]. For this reason, new therapeutic options are urgently needed to provide more effective and safe treatment, and several researchers have focused on the use of complementary and alternative medicines, such as natural products or traditional herbs. However, reliable data regarding the efficacy and safety of such treatments are lacking [5].

Our novel formula KIOM-MA-128 (K-M-128) was generated by fermenting KIOM-MA using probiotics (*Lactobacillus rhamnosus)*. KIOM-MA is composed of *Glycyrrhizae Radix (*the roots and rhizomes of *Glycyrrhiza uralensis)*, *Polygoni Cuspidati Rhizoma (*the root of *Polygonum cuspidatum Sieb. et Zucc.*), *Sophorae Flavescentis Radix (*the root of *Sophora flavescens Ait.)*, *Cnidii Rhizoma (*the rhizome of *Cnidium officinale Makino)*, *Arctii Fructus (*the dried fruit of *Arctium lappa L.)*, *Ginseng Radix Alba (*the root of *Panax ginseng C. A. Meyer)*, *Scrophulariae Radix (*the root of *Scrophularia ningpoensis Hemsl.)*, *Zizyphi Semen (*the seeds of *Zizyphus spinosa Hu)*, *Angelica Gigantis Radix (*the root of *Angelica gigas Nakai)*, and *Saposhnikovia Radix (*the root of *Saposhnikovia divaricata Schischkin)*, which have all long been used in natural pharmaceutical treatments in Asia. Previous studies have shown that the non-fermented formula, KIOM-MA, has anti-inflammatory and anti-allergic effects [6, 7]. Furthermore, we have found that K-M-128, the bioconversion product, has anti-atopic dermatitis effects as well as anti-cancer effects [7, 8]. However, the intracellular mechanism underlying the anti-allergic effects remains unclear, although we have elucidated that K-M-128 inhibits an antigen/IgE-induced response through the reduction of cPLA_2_, COX-2 and other signaling molecules [9]. In recent research, K-M-128 prevented against IL-6 induced intestinal barrier disruption via the regulation of tight junction proteins in a colon cancer cell line [10]. Therefore, we investigated whether K-M-128 can regulate acute colitis in vivo. To accomplish this aim, we investigated the effects of K-M-128 in a DSS-induced mouse colitis model and evaluated whether they underlie the protective effects of this formula against colitis, inflammation, and damage to tight junctions in colonic crypts.

## Methods

### Preparation of KIOM-MA-128

The preparation of K-M-128 has been previously described [6, 11]. Briefly, the KIOM-MA formula (*Glycyrrhizae Radix*, *Polygoni Cuspidati Rhizoma*, *Sophorae Flavescentis Radix*, *Cnidii Rhizoma*, *Arctii Fructus*, *Ginseng Radix Alba*, *Scrophulariae Radix*, *Zizyphi Semen*, *Angelica Gigantis Radix*, and *Saposhnikovia Radix*) was purchased from the Korea Medical Herbs Association (Yeongcheon, Korea), and the identification was confirmed by Porf. KiHwan Bae (The College of Pharmacy, Chungnam National University, Daejeon, Korea) [7, 11]. A total of 1.84 kg of this formula (the ratios of each constituent of KIOM-MA are shown in Table 1) was placed in 18.4 L of water and then extracted by boiling for 3 h at 115 °C. The fermentation process was conducted such that the autoclaved KIOM-MA extract was added to the 1% broth media including *Lactobacillus rhamnosus* (1X10^8^ CFU/ml) at 37 °C for 48 h under micro-aerobic conditions followed by filtration through a 60 μm nylon filter (Millipore, Bedford, MA, USA), and the yield was 20.44%. After fermentation, the supernatant of K-M-128 was filtered and then freeze dried. The collected K-M-128 powder was kept at −20 °C until use. The freeze-dried powder was dissolved in saline, and its solution was prepared before every oral administration.

**Table 1:** The weight ratios of each constituent in KIOM-MA-128

No	Name	Ratio by weight
1	*Glycyrrhizae Radix*	1
2	*Polygoni Cuspidati Rhizoma*	1
3	*Sophorae Flavescntis Radix*	2
4	*Cnidii Rhizoma*	1
5	*Arctii Fructus*	2
6	*Ginseng Radix Alba*	2
7	*Scrophulariae Radix*	2
8	*Zizyphi Semen*	1
9	*Angelica Gigantis Radix*	1
10	*Saposhnikovia Radix*	1

### Chemicals and reagents

Dextran sodium sulfate was purchased from MP Biomedicals (Santa Ana, CA, USA), and hematoxylin and eosin solutions were obtained from Sigma Aldrich (St Louis, MO, USA). The ELISA kits for the detection of mouse tumor necrosis factor-alpha (TNF-α) and interleukin-6 (IL-6) were purchased from eBioscience (San Diego, CA, USA), and RIPA lysis buffer was obtained from Millipore (Darmstadt, Germany). Phosphatase and protease inhibitor cocktails were purchased from Roche (Basel, Swiss). A BCA protein quantification kit, fluorescence-tagged antibody and anti-Zonula occludens-1 (ZO-1) antibody were obtained from Thermo Fisher Scientific (Waltham, MA, USA), and anti-F4/80 antibody was purchased from Santa Cruz Biotechnology (Dallas, TX, USA).

### Animals

Male ICR mice (6 weeks) were purchased from Samtako Inc. (Osan, Korea), divided into 4 groups including 6 mice per group under specific pathogen-free conditions (21–24 °C and 40–60% relative humidity) with a 12 h light/dark cycle, and provided with standard rodent food (Orientbio Inc., Sungnam, Korea) and water. All procedures for the animal study were approved by the Korea Institute of Oriental Medicine Institutional Animal Care and Use Committee (KIOM-IACUC) and were conducted in accordance with US guidelines (NIH publication #83–23, revised in 1985).

### Induction of DSS-induced colitis

After acclimation, the mice were orally administered (oral gavage) saline with K-M-128 (125 or 250 mg/kg). The treatment of DSS was provided with or without 5% DSS-containing water ad libitum for 6 days; then, the mice were provided DSS-free water for an additional 2 days. The mice were sacrificed with CO_2_ gas at the end of the experiment; the colon lengths were measured, and proteins were extracted from the colonic tissues.

### Measurements of body weight, colon length, and disease activity index (DAI)

During the experimental schedule, the mouse body weights and the disease activity index (DAI) were measured daily before the oral administration of K-M-128. The colonic lengths were measured according to photographs after the animals were sacrificed. The DAI was determined as the sum of the diarrheal and bloody stool scores [12]. The DAI scoring system is described in Table 2.

**Table 2:** Disease activity index (DAI) scoring system. The score was determined based on the characteristics of two stool types, and the sum of the scores of two parameters was defined as the DAI score

Score	Diarrheal stool score	Bloody stool score
0	Normal stool	Normal colored stool
1	Mildly soft stool	Brown stool
2	Very soft stool	Reddish stool
3	Watery stool	Bloody stool

### Large-intestine endoscopy and histological analysis

On days 5 and 8 of the experiment, we investigated the colons of mice anesthetized with isoflurane by endoscopy. Endoscopies in all mice were performed using a mini-endoscope (670 mm length and 2.8 mm diameter, OLYMPUS, Shinjuku-ku, Tokyo, Japan) with a visible light source, and high-resolution images were obtained. After the endoscopy procedure on day 8, whole blood was collected from the abdominal vein of mice, and the animals were then killed for tissue collection. The isolated colons were fixed with 4% paraformaldehyde solution, embedded in paraffin block, and sectioned using a microtome. Histological sections were stained with a hematoxylin and eosin solution or incubated with antibodies to detect F4/80, a macrophage marker, and ZO-1, a tight junction protein.

### Enzyme-linked immunosorbent assay for IL-6 and TNF-α

Collected whole blood was left at 4 °C overnight and then centrifuged (3000×g at 4 °C) for 15 min. The separated serum was stored at −80 °C until use. The levels of IL-6 and TNF-α in the serum were determined by ELISA kits according to the manufacturer’s instructions.

### Immunoblotting analysis

Mouse intestinal protein was extracted with RIPA lysis buffer with phosphatase and protease inhibitor cocktails. After protein quantification with a BCA kit, the levels of ZO-1 in the protein lysates were analyzed by immunoblotting analysis using anti-ZO-1 antibody (220 kDa). Briefly, proteins of equal amount (20 μg/20 μl) were separated by 8% and 12% SDS-PAGE gels and then transferred to PVDF membranes. The membranes were incubated with a 1:200 dilution of ZO-1 antibody at 4 °C overnight. The following day, the membranes were incubated with secondary antibodies and detected using the ChemiDoc Touch Imaging System (Bio-Rad, Hercules, CA, USA).

### Statistical Analysis

All statistical analyses were performed with SPSS version 18, and graphs were drawn with GraphPad Prism version 5. Experimental values are given as the means ± SEM. The significant difference was determined by one-way ANOVA test. *p* values less than 0.05 were regarded as statistically significant.

## Results

### DSS-induced colonic disorder was reduced after treatment with KIOM-MA-128.

**Fig. 1:**
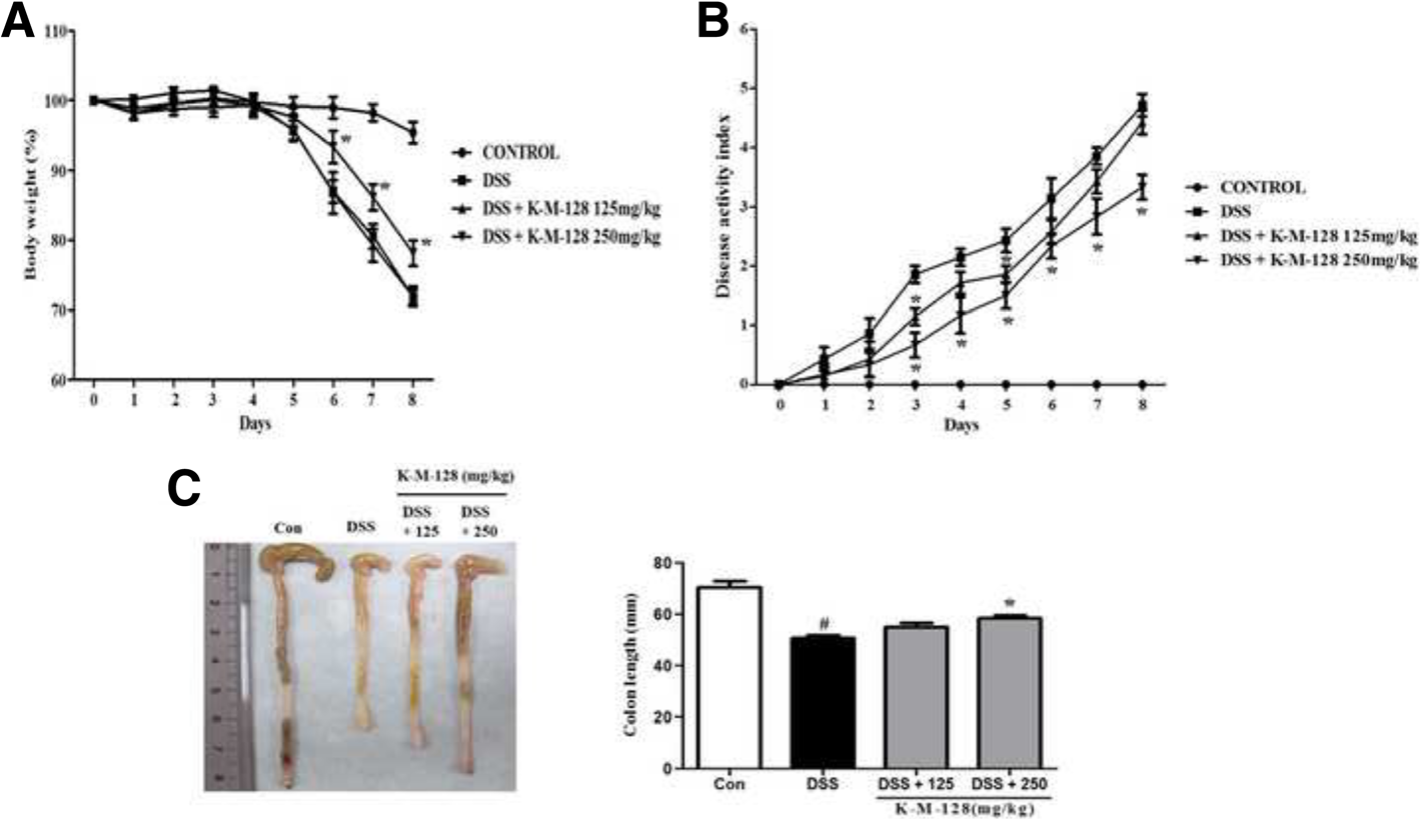
The effects of KIOM-MA-128 on morphological changes in a DSS-induced colitis model. Mice were orally administered K-M-128 (125 or 250 mg/kg) before DSS treatment. The body weights (**a**) and DAI scores (**b**) were monitored before K-M-128 treatment during the experimental schedule. The colonic lengths (**c**) of mice were measured after the colons were isolated from the sacrificed mice. The results represent the mean ± SEM values of each mouse in the same group. # *p* < 0.05 versus the control group, * *p* < 0.05 versus the DSS-treated group

We first examined the effects of our formula, K-M-128, on the intestinal phenotype in a mouse model. K-M-128 reduced the loss of body weight and DAI scores associated with DSS treatment in a dose-dependent manner (Fig. 1a and b). Moreover, the abnormally short colon lengths induced by DSS were absent in mice treated with our formula (Fig. 1c). These results demonstrate that our formula attenuates the symptoms of DSS-induced colitis in a mouse model.

### KIOM-MA-128 has a therapeutic effect in a mouse colitis model.

**Fig. 2:**
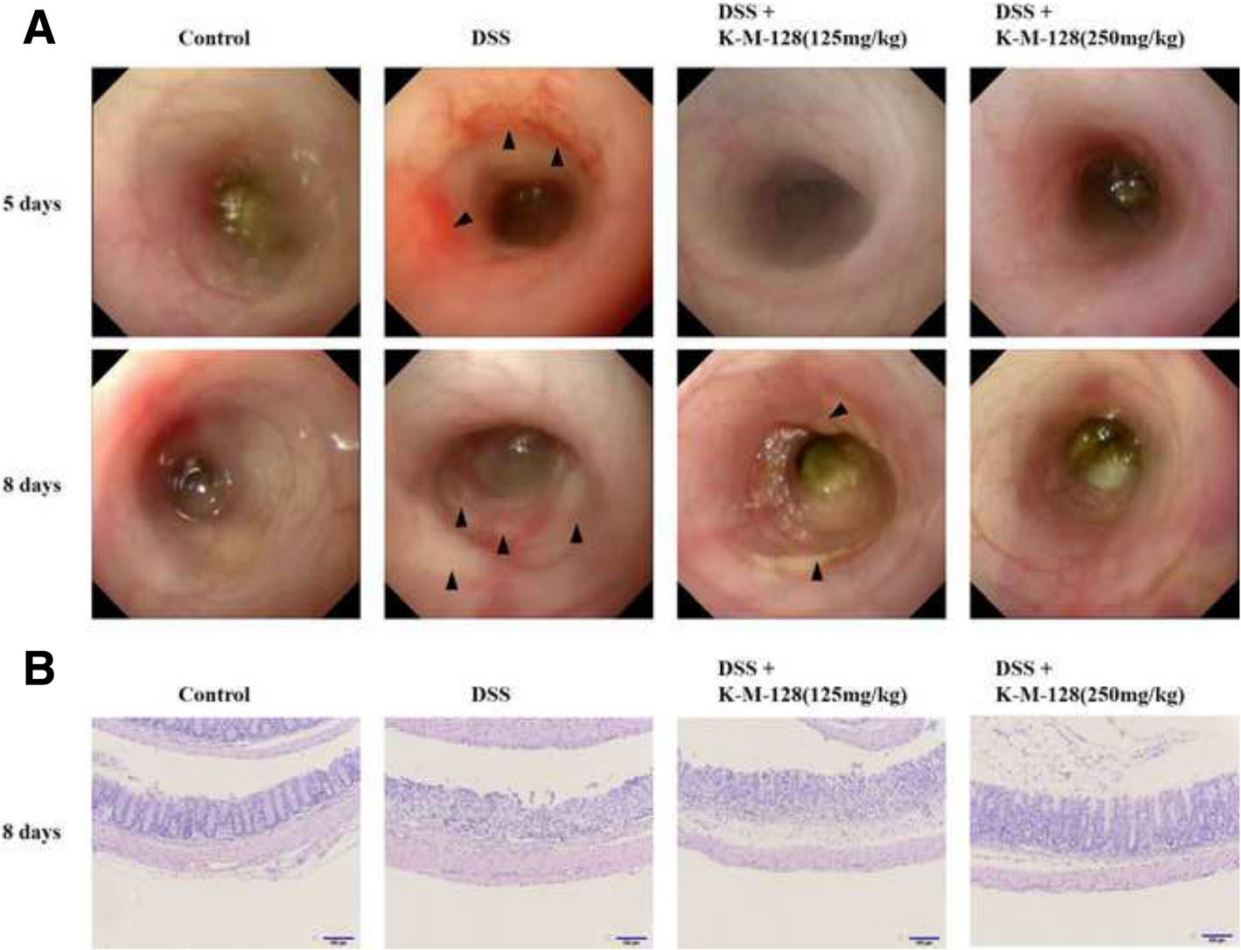
The protective effect of KIOM-MA-128 against colitis in the intestinal crypts. We showed that our formula protected against DSS-induced mucosal damage, as shown by endoscopy (**a**) and hematoxylin & eosin staining (**b**). The *arrow head* indicates the lesion of ulceration

To determine the extent of colitis and the effects of our formula, we examined the histological changes in the large intestine after the administration of DSS with K-M-128. Endoscopy is essential for diagnosing and treating IBD, including CD and UC. The technique is used to diagnose and distinguish among the diseases and to observe the effects of therapeutic approaches [13, 14]. As shown in Fig. 2a, we obtained images of DSS-induced damage in colon tissues, and K-M-128 protected against this damage, as viewed with a colonic mini-endoscope at days 5 and 8. Moreover, DSS-mediated damage of colonic crypts was prevented by treatment with K-M-128 in a dose-dependent manner (Fig. 2b). These results suggest that our novel formula has a therapeutic effect against DSS-induced colitis.

### KIOM-MA-128 inhibited the DSS-induced inflammatory mechanism.

**Fig. 3:**
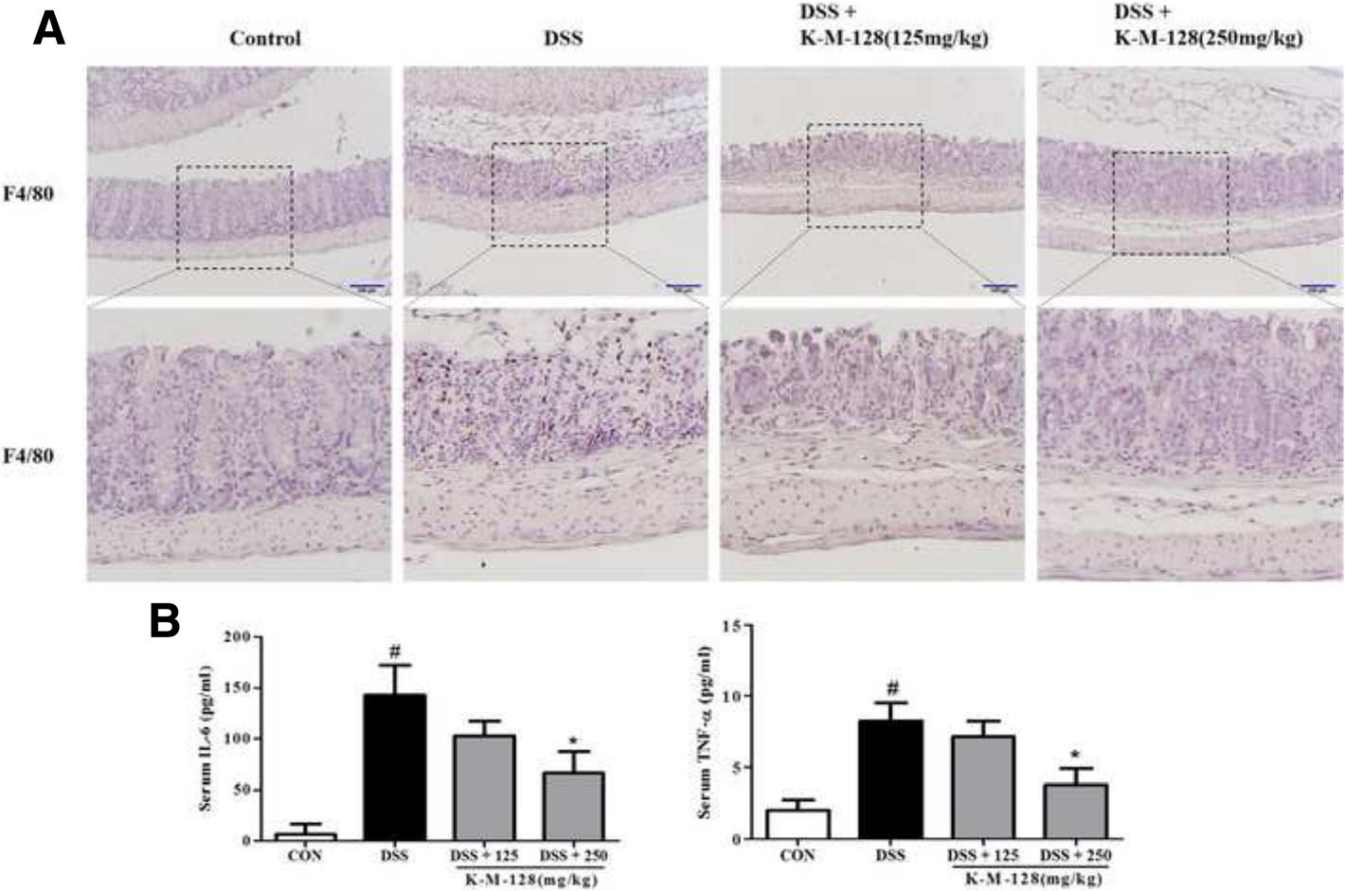
The changes in DSS-induced inflammatory responses after KIOM-MA-128 treatment. K-M-128 repressed macrophage infiltration in the mucosa at 8 days (**a**) and secreted cytokines into the serum (**b**) after DSS treatment. Cytokine results represent the mean ± SEM values of each mouse in the same group. # *p* < 0.05 versus the control group, * *p* < 0.05 versus the DSS-treated group

According to our previous study, we found that our formula has anti-colitis potential by examining intestinal characteristics. We investigated the activation of the inflammatory cascade in the DSS-induced colitis model and found that macrophage infiltration and cytokine secretion are involved in inflammatory signaling. Our results showed that macrophages were recruited to the mucosa after DSS treatment, whereas K-M-128 repressed the macrophage infiltration and the secretion of TNF-α and IL-6 after DSS treatment (Fig. 3a and b). Based on the above findings, K-M-128 possesses anti-inflammatory properties, regulating macrophage infiltration and cytokine production in acute colitis.

### KIOM-MA-128 represses the disruption of intestinal tight junctions.

**Fig. 4:**
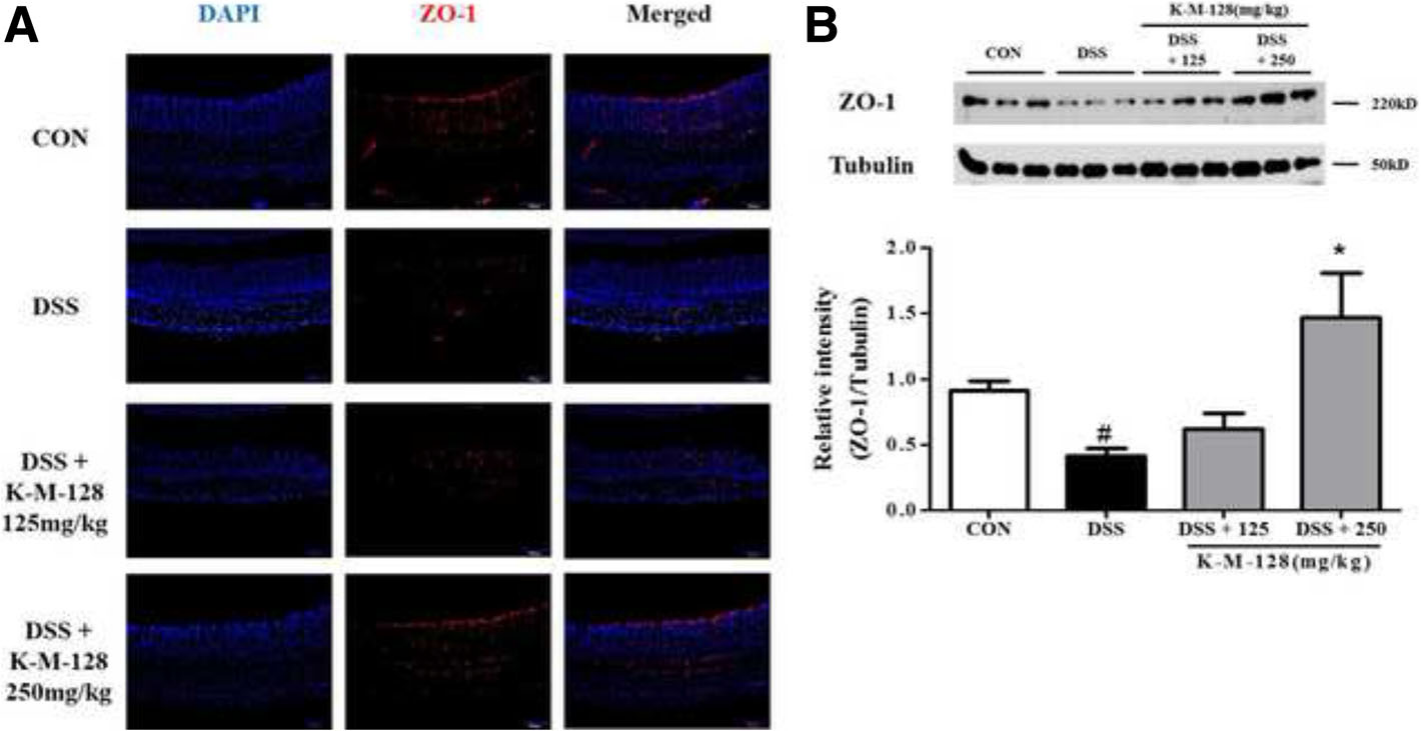
The protective effect of KIOM-MA-128 against the degeneration of tight junctions. We stained the ZO-1 protein expressed in the intestinal crypts, and our formula repressed the degradation of ZO-1 after DSS treatment (**a**). In tissue lysates, our formula attenuated the down-regulation of ZO-1 after DSS treatment (**b**). These western blot data represent the mean ± SEM values of each mouse in the same group. # *p* < 0.05 versus the control group, * *p* < 0.05 versus the DSS-treated group

Because our data indicated that K-M-128 treatment suppresses DSS-induced colitis, it will be of interest to investigate whether this effect is related to colonic tight junctions. ZO-1 expression was decreased after DSS administration in colonic crypts, and this down-regulation was blocked by treatment with K-M-128 in a dose-dependent manner (Fig. 4a). Moreover, DSS decreased ZO-1 protein expression, and this expression was restored by K-M-128 treatment (Fig. 4b). Together, our results show that K-M-128 represses the down-regulation of tight junction proteins in an acute colitis model, and K-M-128 might be involved in anti-colitis mechanisms in DSS-induced colitis.

## Discussion

UC is a colonic inflammation caused by several factors, and chemical-induced animal models are useful to discover the mechanism and to establish novel clinical approaches in colitis treatment. Patients with UC account for half of all patients with IBD in the United States, and the symptoms of UC can be improved through various treatments. However, these treatments have numerous side effects, and drug tolerance is observed in some patients [15, 16]. Therefore, a new therapeutic method remains to be developed and is currently needed.

Administering DSS in the drinking water to mice or other animals for several days has direct toxic effects on epithelial cells in the intestinal crypts and on the integrity of the barrier, recapitulating the major symptoms of acute and chronic colitis. Therefore, the DSS-induced colitis model is useful for researching the contributions of immune mechanisms in colitis. Previous studies have shown that DSS administration for 7 or 10 days induces acute colitis, a loss of body weight, shortening of the colon, inflammatory gene expression, and phenotypes of histological staining [17, 18]. Therefore, we induced acute colitis by DSS administration in the drinking water for 6 days in ICR mice. Then, we confirmed the typical symptoms of UC, such as loss of body weight, intestinal shortening, and crypt damage, and found that these factors were reduced by treatment with K-M-128. Therefore, our formula suppresses the onset of UC.

Then, we identified inflammatory mechanisms in the colitis model. Macrophage infiltration and cytokine secretion have major roles in intestinal inflammation; macrophages and cytokines control multiple aspects of the inflammatory response. Macrophages have a crucial role in innate and adaptive immune responses. F4/80, which recognizes a murine macrophage surface protein, has been used to detect macrophage infiltration in the immune response. In our data, macrophages recognized by F4/80 were widely distributed in colon tissues, and the levels of inflammatory cytokines such as TNF-α, IL-6 and IL-1β were elevated in the sera of DSS-treated mice. In support of this finding, recruited macrophages are known to be associated with the secretion of TNF-α, IL-6 and IL-1β [19]. Therefore, TNF-α, IL-6 and IL-1β are very important cytokines in IBD because their levels are increased in the mucosa and serum in IBD, including UC and CD. For this reason, many studies have examined the regulation of cytokines as potential targets in therapeutic approaches [16, 20]. In the present study, we confirmed that DSS treatment induced immune responses such as macrophage infiltration and cytokine secretion, whereas K-M-128 repressed the pro-inflammatory cascade. Our data indicated that the mechanism of K-M-128 was related to the reduction of the macrophage infiltration and cytokine secretion from the mucosa into the serum.

We anticipated that these inflammatory responses would be associated with the barrier function of intestinal crypts. The intestinal epithelial barrier has important protective effects against external insults such as antigens, toxins, or infections [21]. Several studies have suggested that the down-regulation of occludin, one of the tight junction proteins, is associated with IBD such as UC and CD [22, 23]. In addition, tight junction protein-1, also known as ZO-1, is associated with intracellular tight junctions. Thus, when DSS induces acute colitis, ZO-1 expression is also reduced, and intestinal permeability is subsequently increased [24, 25]. Therefore, the regulation of ZO-1 expression might be important in the treatment and prevention of colitis diseases. In this study, we found that the integrity of epithelial crypts relies on paracellular tight junctions through the expression of ZO-1 protein. If our formula can restore damaged epithelial mucosa, it might be associated with tight junction proteins. As shown in our results, K-M-128 prevented the down-regulation of tight junctions and the damage of intestinal crypts. ZO-1 expression in the K-M-128 (250 mg/kg) group was elevated compared with the control group. These results might indicate that K-M-128 had a better effect on intestinal barrier function. Moreover, our formula restored the barrier permeability dysfunction caused by IL-6 [10].

## Conclusion

We conclude that our novel formula K-M-128 has anti-colitis effects through the regulation of paracellular tight junctions. Therefore, K-M-128 suppresses the inflammatory response by inhibiting the penetration of pro-inflammatory factors into the intestinal mucosa. K-M-128 might be further developed as an effective preventive approach to treat intestinal inflammation.

## References

[1] Baumgart DC, Sandborn WJ (2007). Inflammatory bowel disease: clinical aspects and established and evolving therapies. Lancet.

[2] Wang G, Ren J, Liu S, Chen J, Gu G, Wang X, Fan C, Li J (2012). Clinical characteristics of non-perianal fistulating Crohn's disease in China: a single-center experience of 184 cases. Chin Med J.

[3] Hanauer SB (2006). Inflammatory bowel disease: epidemiology, pathogenesis, and therapeutic opportunities. Inflamm Bowel Dis.

[4] Bouma G, Strober W (2003). The immunological and genetic basis of inflammatory bowel disease. Nat Rev Immunol.

[5] Langmead L, Rampton D (2006). Review article: complementary and alternative therapies for inflammatory bowel disease. Aliment Pharmacol Ther.

[6] Oh Y-C, Cho W-K, Jeong YH, Im GY, Kim A, Hwang Y-H, Kim T, Song KH, Ma JY (2012). A Novel Herbal Medicine KIOM-MA Exerts an Anti-Inflammatory Effect in LPS-Stimulated RAW 264.7 Macrophage Cells. Evidence-Based Complementary and Alternative Medicine.

[7] Chung TH, Kang TJ, Cho W-K, Im GY, Lee GS, Yang MC, Cho C-W, Ma JY (2012). Effectiveness of the novel herbal medicine, KIOM-MA, and its bioconversion product, KIOM-MA128, on the treatment of atopic dermatitis. Evidence-Based Complementary and Alternative Medicine 2012.

[8] Kim A, Im M, Yim N-H, Hwang Y-H, Yang HJ, Ma JY (2015). The novel herbal cocktail MA128 suppresses tumor growth and the metastatic potential of highly malignant tumor cells. Oncol Rep.

[9] Park KI, Kim DG, Yoo JM, Ma JY (2016). The Herbal Medicine KIOM-MA128 Inhibits the Antigen/IgE-Mediated Allergic Response in Vitro and in Vivo. Molecules.

[10] Park KI, Kim DG, Lee BH, Ma JY (2016). Fermented Herbal Formulas KIOM-MA128 Ameliorate IL-6-Induced Intestinal Barrier Dysfunction in Colon Cancer Cell Line. Mediat Inflamm.

[11] Kim A, Ma JY (2014). Anti-melanogenic activity of the novel herbal medicine, MA128, through inhibition of tyrosinase activity mediated by the p38 mitogen-activated protein kinases and protein kinase signaling pathway in B16F10 cells. Pharmacogn Mag.

[12] Tanaka K-I, Namba T, Arai Y, Fujimoto M, Adachi H, Sobue G, Takeuchi K, Nakai A, Mizushima T (2007). Genetic evidence for a protective role for heat shock factor 1 and heat shock protein 70 against colitis. J Biol Chem.

[13] Daperno M, Sostegni R, Lavagna A, Crocella L, Ercole E, Rigazio C, Rocca R, Pera A (2004). The role of endoscopy in inflammatory bowel disease. Eur Rev Med Pharmacol Sci.

[14] Becker C, Fantini M, Wirtz S, Nikolaev A, Kiesslich R, Lehr H, Galle P, Neurath M (2005). In vivo imaging of colitis and colon cancer development in mice using high resolution chromoendoscopy. Gut.

[15] Mizoguchi A (2011). Animal models of inflammatory bowel disease. Prog Mol Biol Transl Sci.

[16] Colitis–Pathophysiology U: Inflammatory bowel disease part I: ulcerative colitis–pathophysiology and conventional and alternative treatment options. *Alternative medicine review* 2003, 8(3):247–283. 12946238

[17] Chassaing B, Aitken JD, Malleshappa M, Vijay-Kumar M. Dextran Sulfate Sodium (DSS)-Induced Colitis in Mice. Curr Protoc Immunol 2014:15.25. 11-15.25. 14. 10.1002/0471142735.im1525s104 PMC3980572 24510619

[18] Wirtz S, Neufert C, Weigmann B, Neurath MF (2007). Chemically induced mouse models of intestinal inflammation. Nat Protoc.

[19] McKnight AJ, Macfarlane AJ, Dri P, Turley L, Willis AC, Gordon S (1996). Molecular cloning of F4/80, a murine macrophage-restricted cell surface glycoprotein with homology to the G-protein-linked transmembrane 7 hormone receptor family. J Biol Chem.

[20] Reinecker H, Steffen M, Witthoeft T, Pflueger I, Schreiber S, MacDermott R, Raedler A (1993). Enhanced secretion of tumour necrosis factor-alpha, IL-6, and IL-1 beta by isolated lamina propria mononuclear cells from patients with ulcerative colitis and Crohn's disease. Clin Exp Immunol.

[21] Groschwitz KR, Hogan SP (2009). Intestinal barrier function: molecular regulation and disease pathogenesis. J Allergy Clin Immunol.

[22] Poritz L, Lynch C, Tilberg A, Khin S, Page M, Koltun W. Decreased expression of occludin in the intestine of patients with inflammatory bowel disease. In: FASEB JOURNAL: 1997: FEDERATION AMER SOC EXP BIOL 9650 ROCKVILLE PIKE, BETHESDA, MD 20814-3998 USA; 1997: 1800-1800.

[23] Kucharzik T, Walsh SV, Chen J, Parkos CA, Nusrat A. Neutrophil transmigration in inflammatory bowel disease is associated with differential expression of epithelial intercellular junction proteins. Am J Pathol. 2001;159(6):2001–9. 10.1016/S0002-9440(10)63051-9 PMC1850599 11733350

[24] Stevenson BR, Siliciano JD, Mooseker MS, Goodenough DA (1986). Identification of ZO-1: a high molecular weight polypeptide associated with the tight junction (zonula occludens) in a variety of epithelia. J Cell Biol.

[25] Poritz LS, Garver KI, Green C, Fitzpatrick L, Ruggiero F, Koltun WA (2007). Loss of the tight junction protein ZO-1 in dextran sulfate sodium induced colitis. J Surg Res.

